# Simultaneous transcranial and transcutaneous spinal direct current stimulation to enhance athletic performance outcome in experienced boxers

**DOI:** 10.1038/s41598-021-99285-x

**Published:** 2021-10-05

**Authors:** Ali-Mohammad Kamali, Milad Kazemiha, Behnam Keshtkarhesamabadi, Mohsan Daneshvari, Asadollah Zarifkar, Prasun Chakrabarti, Babak Kateb, Mohammad Nami

**Affiliations:** 1 Neuroscience Laboratory, NSL (Brain, Cognition and Behavior), Department of Neuroscience, School of Advanced Medical Sciences and Technologies, Shiraz University of Medical Sciences, Shiraz, Iran; 2 DANA Brain Health Institute, Iranian Neuroscience Society-Fars Chapter, Shiraz, Iran; 3 Neuroscience Center, Instituto de Investigaciones Científicas y Servicios de Alta Tecnología (INDICASAT AIP), City of Knowledge, Panama City, Panama; 4 Department of Physiology, School of Medicine, Shiraz University of Medical Sciences, Shiraz, Iran; 5 Techno India NJR, Institute of Technology, Udaipur, 313003 Rajasthan India; 6 Brain Mapping Foundation and Society for Brain Mapping and Therapeutics, Los Angeles, CA USA; 7 Brain, Cognition and Behavior Unit, Department of Neuroscience, School of Advanced Medical Sciences and Technologies, Shiraz University of Medical Sciences, Shiraz, Iran

**Keywords:** Cognitive neuroscience, Motor control

## Abstract

Transcranial direct current stimulation (tDCS) is among the rapidly growing experimental approaches to enhance athletic performance. Likewise, novel investigations have recently addressed the effects of transcutaneous spinal Direct Current Stimulation (tsDCS) on motor functions such as reduced reaction time. The impact of tDCS, and tsDCS might be attributed to altered spontaneous neural activity and membrane potentials of cortical and corticomotoneuronal cells, respectively. Given the paucity of empirical research in non-invasive brain stimulation in sports neuroscience, especially in boxing, the present investigation studied the effects of neuromodulation on motor and cognitive functions of professional boxers. The study sample comprised 14 experienced male boxers who received random sequential real or sham direct current stimulation over the primary motor cortex (M1) and paraspinal region (corresponding to the hand area) in two sessions with a 72-h interval. Unlike sham stimulation, real stimulation improved selective attention and reaction time of the experienced boxers [enhanced selective attention (*p* < 0.0003), diminished right hand (*p* < 0.0001) and left hand reaction time (*p* < 0.0006)]. Meanwhile, the intervention left no impact on the participants’ cognitive functions (*p* > 0.05). We demonstrated that simultaneous stimulation of the spinal cord and M1 can improve the performance of experienced boxers through neuromodulation. The present study design may be extended to examine the role of neurostimulation in other sport fields.

## Introduction

The three attributes of ‘being faster, more agile, and stronger in athletic performance’ are perhaps regarded as key pillars in most competitive sports. In recent years, there has been an increasing interest among researchers to cross-link sport science with neuroscience and use non-pharmacological brain stimulation approaches including neuromodulation to enhance athletic performance^1^. Transcranial direct current stimulation (tDCS) is a non-invasive technique in which weak direct current leads to changes in the brain excitability.

In that realm, Davis coined the term “neurodoping”, which refers to the use of new techniques to enhance physical and mental performance in athletes^2^. Some earlier reports have also substantiated that non-invasive brain stimulation techniques may potentially increase motor learning, muscular strength as well as specific motor skills, and decrease level of fatigue and perceived exersion^3^. In practice, tDCS transmits a weak (1–2 mA) constant current through electrodes placed on one’s scalp for 5–20 min. Part of this electrical current that is transmitted to the brain tissue is known to affect the neuronal excitability, action potential threshold, and subsequent changes through neuroplasticity^4^.

Some recent investigations have demonstrated enhance athletic performance through tDCS. Namely, anodal stimulation over the temporal cortex (TC) has been found to reduce the perceived exertion and heart rate and improve the overall performance in professional cyclists. The stimulation could improve their peak power output (PPO: the highest power that a cyclist can maintain cycling for more than 1 min) by 4%^5^.

In another report, authors observed that simultaneous stimulation of the motor cortex (leg area) and left temporal cortex significantly improves athletic performance indicators for the strength and endurance variables^6^.

In an earlier research, out team showed that simultaneous inhibition of the dorsolateral prefrontal cortex (dlPFC) inhibition and stimulation of the cerebellar cortex increase the accuracy of performance in professional pistol shooters. Indeed, our intervention increased the mean shooting score of the experienced pistol shooters by 2.3%. However, the stimulation could not leave an effect on the athletes’ shooting speed/delay. Additionally, tDCS reduced the number of task-specific tremors which potentially supports the theatrical relationship between tDCS-induced reduced physiological tremor and enhanced shooting performance^7^.

Transcutaneous Spinal Direct Current Stimulation (tsDCS) is another non-invasive central nervous system (CNS) stimulation technique. Many studies have confirmed the effectiveness of anodal tDCS over M1 in improving motor learning in healthy individuals^8–10^, whereas tsDCS studies have mainly focused on patients^11,12^ and not healthy individuals. According to a recent study, anodal tsDCS was shown to improve motor unit recruitment^13^. Given the above evidence, it can be hypothesized that the integration of tDCS and tsDCS techniques may additively or synergistically enhance the athletic performance.

Accuracy, agility, and endurance are of great importance in boxing. However, to our best knowledge, no study has so far investigated the effects of tDCS and tsDCS techniques in boxers. Moreover, simultaneous stimulation of athletes’ brain and spinal cord has not been performed in any study. In boxing, an athlete needs to punch his/her opponent, hence the number of clean punches in boxing is the main scoring criteria. Head is the main target of boxers, because punches to head can lead to serious injuries and knockout, and as a result the referee may stop the match. The reaction speed, accuracy, and visuospatial working memory of athletes can significantly affect the result of a boxing match. While the modulation of these factors appears to collectively affect the boxer’s performance, research on the effect of neurostimulation in boxing is lacking. Therefore, this study hypothesized that a simultaneous brain and spinal cord stimulation is effective in improving the boxers’ motor and cognitive performance.

## Materials and methods

### Participants

This was a factorial single-armed randomized trial in which subjects were assigned to sham or true tDCS + tsDCS intervention through simple randomization in 1:1 ratio. The ethical approval for this study was obtained from the Shiraz University of Medical Sciences (SUMS) (No. 98-01-74-21827). All methods were performed in accordance with the relevant guidelines and regulations in line with the declaration of Helsinki.

The entire process including its rationale and objective, the participants' role and safety consideration were explained to each candidate in plain language. The participants were then asked to sign a written informed consent indicating that their data would remain confidential and they may resign from the process on their discretion whenever during the project. The consent was made in two identical copies of which the participants could retain one.

The study included professional male boxers who had at least 2 years of consistent boxing exercise. With regards to the training hours, participants had 3 times training (roughly 6 h in total) per week. Case selection followed a convenient cluster random sampling method, whereby 14 skilled boxers from the city of Shiraz were enrolled. This study refers to amateur boxing (Olympic Boxing) as a variant of boxing widely practiced at the Olympic Games. This style and its rules have been well defined by the Armature International Boxing Association (http://www.aiba.org). Participants were confirmed not to have psychological or neurological disorders. Not only they refrained from tobacco and alcohol for 3 months’ prior to the tests, but also they had not used caffeine-containing substances such as coffee on test days. In addition, all participants reported to have observed our recommended sleep hygiene measures and maintained their routine diet on test days. This study was a single-armed randomized trial and participants sequentially received either sham or real tDCS via simple randomization. Table 1 summarizes the participants’ demographic information.

**Table 1:** Participants’ demographic information (n = 14), mean ± SEM (standard error of mean).

Mean age in years	22.3 ± 3
Mean years of training in boxing	3.2 ± 1.1
Mean years of formal education	15 ± 2
Mean weight (kg)	75 ± 19
Mean height (cm)	176 ± 16

### Experimental design

**Figure 1:**
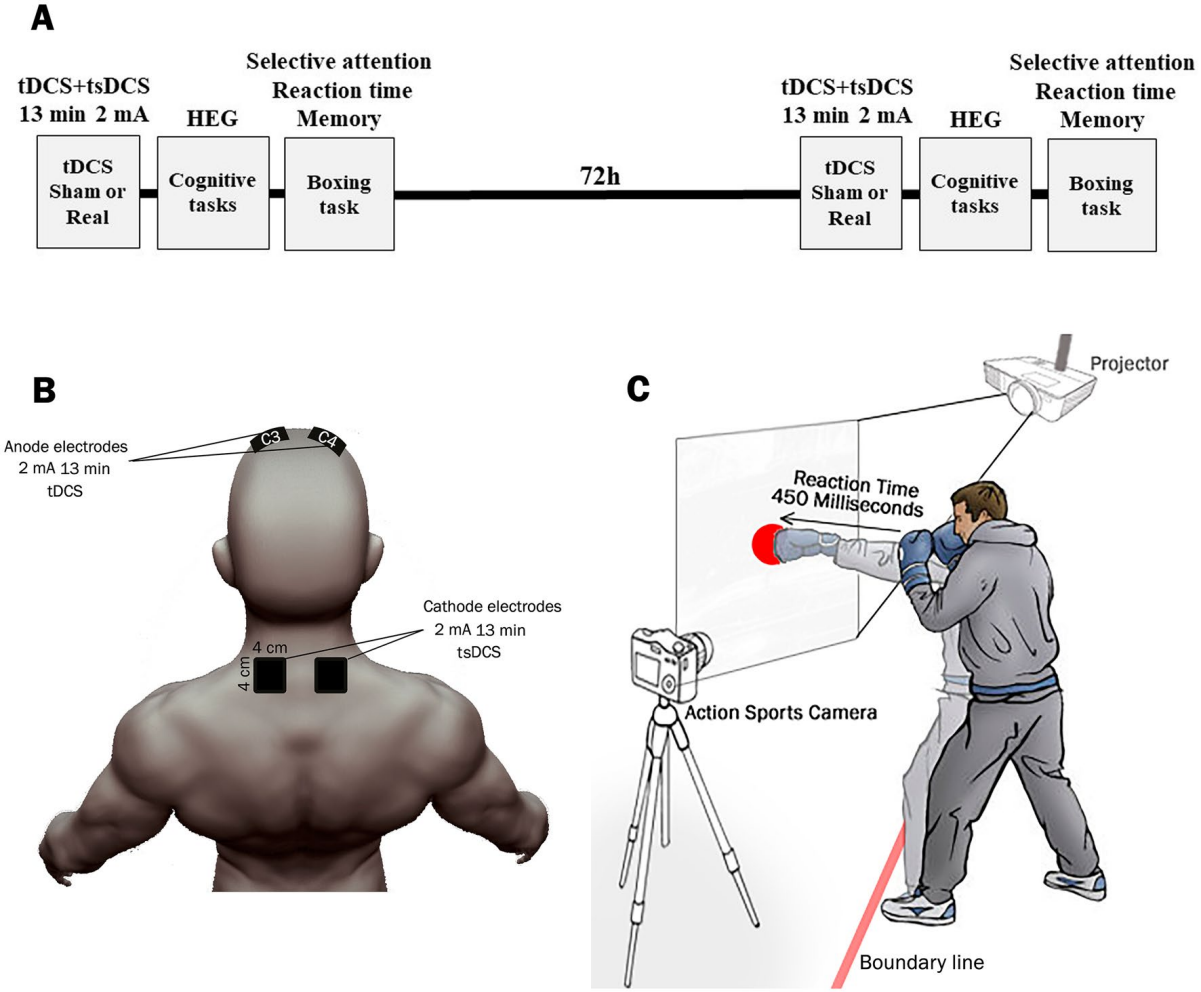
Study protocol, the tDCS + tsDCS montages used for brain stimulation and boxing task. (**A**) Participants were randomly assigned to either sham or real tDCS + tsDCS at 2 mA for 13 min over the first session. Then, they performed 2 tasks including spatial span (short-term memory) and double trouble (response inhibition) from CBS-cognitive platform (see “Materials and methods” section) with the intervals of 2 min’ rest. CBS-CP and the HEG data were concurrently recorded while subjects carried out the tasks. Later, they performed the boxing task and their selective attention, reaction time and Short-term memory were recorded. After 72 h, the real group received sham tDCS + tsDCS whereas sham group received real tDCS for 13 min and they performed the rest of the tasks similar to the first session. (**B**) 2 mA anodal tDCS pad electrodes were placed over the C3 and C4 (M1 hand area) for a course of 13 min. Both cathodal electrodes were placed bilaterally adjacent to spinous processes of C5-T1 and were not overlapping. The size of the electrodes is depicted in the Figure. (**C**) The participants stood at the same distance from the wall every 2 days. Ten circles appeared on the wall with random intervals. The period between the appearance of the circles on the wall and the impact of the boxers' punch on them was considered as reaction time.

This double-blind experiment was done in two sessions 72 h apart. Participants were blinded to session plans and a neuroscientist did the interventions. Consistently, the experimenters remained blinded to the experimenter and participants in terms of the type (sham or real) of stimulation. Participants were randomly assigned to sham or real tDCS in the first session. After 72 h, those who received sham first, received real tDCS in the second session, and vice versa. Following the brain stimulation, boxers were asked to perform 2 tasks from the Cambridge Brain Sciences Cognitive Platform (CBS-CP). Simultaneously, hemodynamic response of left frontopolar region (FP1) was evaluated using the hemoencephalography (HEG). Afterward, the boxer warmed up and performed three maximal contractions in each hand using the handgrip strength evaluation. Boxers were then required to perform three boxing tasks (selective attention, reaction time and visuospatial working memory) (Fig. 1A).

### Transcranial direct current stimulation (tDCS)

Real or sham tDCS was delivered by electric stimulator (Neurostim-2, Medina Teb, Tehran). In each experimental session, saline-soaked sponge coated electrodes (4 * 4 cm^2^) were placed on the regions of interest as described below. Skin sites under electrodes were cleaned up with alcohol. Two anodal electrodes were positioned bilaterally over C3 and C4 area (M1 hand area) based on the international 10–20 EEG electrode placement system, while the two cathodal electrodes were placed bilaterally adjacent to spinous processes of C5-T1 and were not overlapping. In the real session, the current ramped up from 0 to 2 mA in 30 s and maintained constant for 13 min. In the sham session, the sham stimulation followed the same montage of real stimulation while after 30 s, despite ongoing count down and light indicators, the electrical current was turned off automatically (Fig. 1B).

### The Cambridge Brain Science Cognitive Platform (CBS-CP)

Cognitive performance is a substrate of athletic function in many instances^14,15^. To distinguish the positive or negative impact of our neurostimulation protocol on cognitive performance of the participants, cognitive assessment was pursued. To do so, a media-rich computerized online platform addressing three higher-order cognitive components of reasoning, memory and verbal ability was used^16^. Our employed test was the Cambridge Brain Science Cognitive Platform (CBS-CP). From the CBS-CP, spatial span (short-term memory) and double trouble (response inhibition) tasks were chosen to evaluate participants’ performance in memory and attention domains, respectively.

### Frontopolar hemodynamic response

The assessment of cortical hemodynamic changes is an easy-to-use surrogate marker to measure neuronal activity^17^.The hemodynamic changes in the left frontopolar cortical region (FP1) can be measured using hemoencephalography (HEG) response^17^. In our study, this assessment was done to identify local intracranial hemodynamic changes in prefrontal cortex (PFC) using a Hemoencephalography (HEG) device (a peanut near infra-red HEG kit, BIOCOMP Research Institute, Los Angeles, CA). By this means, the optical density in the FP1 area was recorded during the completion of both CBS-CP tasks after either sham or real neurostimulation.

### Handgrip strength (HGS) test

Participants were seated in a proper position (approximately 90° hip/knee) and were asked to complete the maximal isometric handgrip strength test using a dynamometer (SAEHAN DHD-3, MSD Europe bvba). To do the test, the participant’s elbow was flexed at a 90° angle while he performed three maximal contractions alternatively in each hand with a 30 s rest period between each contraction. The mean values among these trials in each hand were recorded for statistical analyses.

### Boxing tasks (selective attention, reaction time and visuospatial short-term memory)

#### Selective attention task

This task was designed to assess the participants' selective attention either after sham or real tDCS. Participants were asked to warm up and perform the boxing attention test. In this test, three different color circles (red, blue and yellow) were projected on the wall in front of the boxers. All participants were right-handed as per the Edinburgh Handedness Inventory (EHI)^18^. They were asked to punch the red circles with their right (dominant) hands and the blue ones with their left (non-dominant) hands. Boxers had to restrain their punches when yellow circles appeared on the wall. The test lasted for 180 s. The colors were displayed randomly and the latency in their presentation was quite unpredictable.

#### Reaction time task

This test was designed separately for each hand. Participants stood at the same distance from the wall in two sessions. Their distance to the wall was defined as per the boxers’ mean body weight and height (Table 1), and remained the same for real tES and sham. Ten circles appeared on the wall with random intervals. The latency between the appearance of the circles on the wall and the impact of the boxers' punch on them was recorded as reaction time.

#### Short-term memory task

At the beginning of the test, 9 circles appeared on the wall. The boxers’ assignment was to pay attention when the circles began flashing in sequence, then punch the circles in the same sequence. The performance was indicated by the highest number of circle positions correctly recalled during the task.

### Data analysis

For the selective attention and short-term memory tasks, the process was filmed (1080p at 60 frames/s), and the number of boxers' errors were calculated off-line for statistical analyses.

To assess the reaction time, the movement trajectories of the players’ hands were manually tracked at 60 frames/s using the Kinovea software (version 0.8.15). The mean values among ten trials in each hand were recorded for statistical analysis (Fig. 1C).

Based on the normality of distribution and homogeneity of variance, parametric and non-parametric statistical tests were employed. A series of paired sample *t*-tests were run to compare the differences between sham and real tDCS in terms of the experiment outcomes.

In addition, Wilcoxon signed-rank test was used to analyze data lacking normal distribution. The differences between the sham and real tDCS sessions were evaluated based on the Mean ± SEM (Standard Error of Mean). The *p* values below 0.05 were considered as statistically significant. The SPSS statistical package (Version 22.0.0) was used for data analyses.

## Results

A total of 14 volunteered professional boxers were conveniently recruited in this study.

Table 2 shows the statistical analyses of data (Table 2).

**Table 2:** Statistical analyses of data (significant; NS: non-significant: *).

	p value	Significant
Reaction time of right hands	0.0001	*
Reaction time of left hands	0.0006	*
Selective attention	0.0003	*
Visuospatial memory	0.7202	NS
Spatial span task	0.6576	NS
Double trouble task	0.0719	NS
Hemodynamic response (spatial span task)	0.5222	NS
Hemodynamic response (double trouble task)	0.2239	NS
Hand grip strength of right hands	0.9806	NS
Hand grip strength of left hands	0.9776	NS

### Reaction time of the right hand

**Figure 2:**
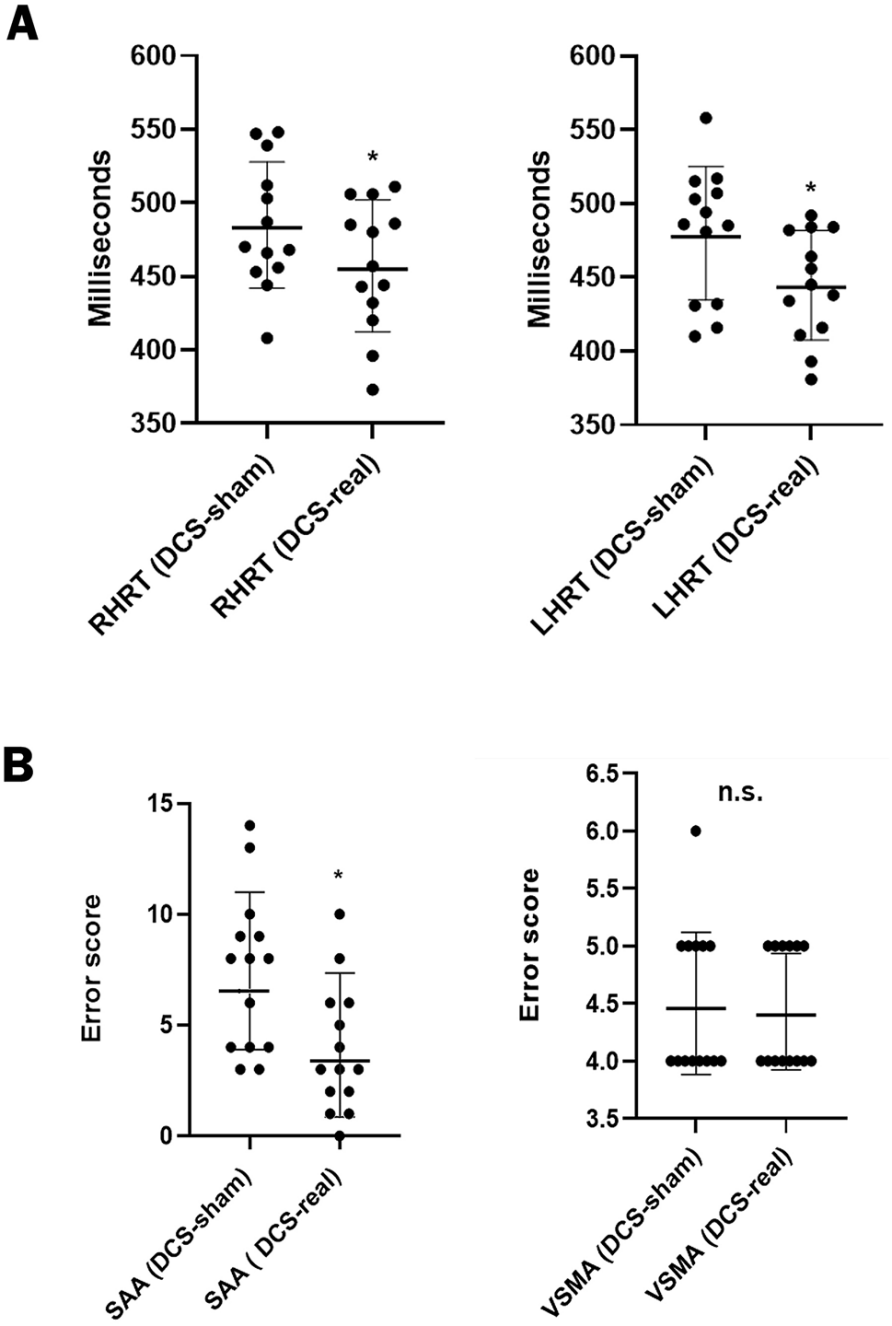
(**A**): Dot plots representing the participants’ performance for the reaction time task (RHRT: Right hand reaction time; LHRT: Left hand reaction time). (**B**): Dot plots representing the participants’ performance for the selective attention and visuospatial memory tasks (SAA.: Selective attention assessment; VSMA.: Visuospatial memory assessment). *Significant; ns: Non-significant. (Selective attention *p* < 0.0003, Right hand reaction time *p* < 0.0001 and Left hand reaction time *p* < 0.0006).

All participants were right-handed. The analysis revealed a statistically significant difference for the right-hand reaction time (RT) between the sham- and real-DCS sessions (Fig. 2A). The real DCS *vs.* sham could decrease the mean RT by 27.9 ms (*p* < 0.0001).

### Reaction time of the left hand

The analysis also revealed a statistically significant difference between the left-hand (non-dominant hand) reaction time of boxers in the sham- and real-DCS sessions (Fig. 2A). The real DCS *vs.* sham was able to reduce the mean RT by 35 ms (*p* < 0.0006).

### Selective attention

The analysis revealed a statistically significant difference in the selective attention score between the sham- and real-DCS sessions (Fig. 2B). Notably, the real DCS *vs.* sham could decrease the mean error scores by 47.5% (*p* < 0.0003) (Fig. 2B).

### Visuospatial working memory

In this assessment, the analysis revealed no statistically significant difference between the sham- and real-DCS sessions (*p* > 0.05) (Fig. 2B).

### Cognitive behavioral assessment

**Figure 3:**
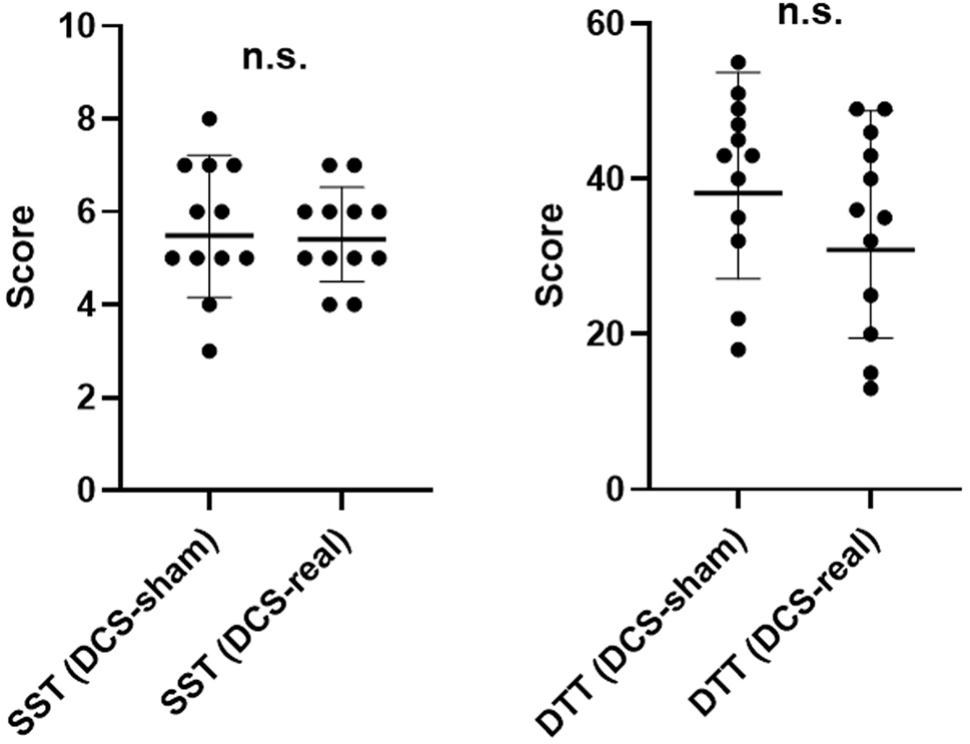
Dot plots representing the participants’ performance for the cognitive behavioral assessment (SST: Spatial span task; DDT: Double trouble task).

According to our findings, there were no statistically significant difference in spatial span and double trouble tasks between the sham- and real-DCS sessions (*p* > 0.05) (Fig. 3).

### Hemoencephalography (HEG) response

A series of paired-sample *t*-tests were used to compare the cerebral blood flow in the FP1 region in the sham and real DCS sessions. Whilst the athletes were doing the double trouble task, their HEG recording revealed no statistically significant increase in FP1 hemodynamic response (*p* > 0.05).

For the spatial span task, the FP1 hemodynamic response was comparable with that of the double trouble task and revealed no statistically significant difference between the sham- and real-DCS sessions (*p* > 0.05).

**Figure 4:**
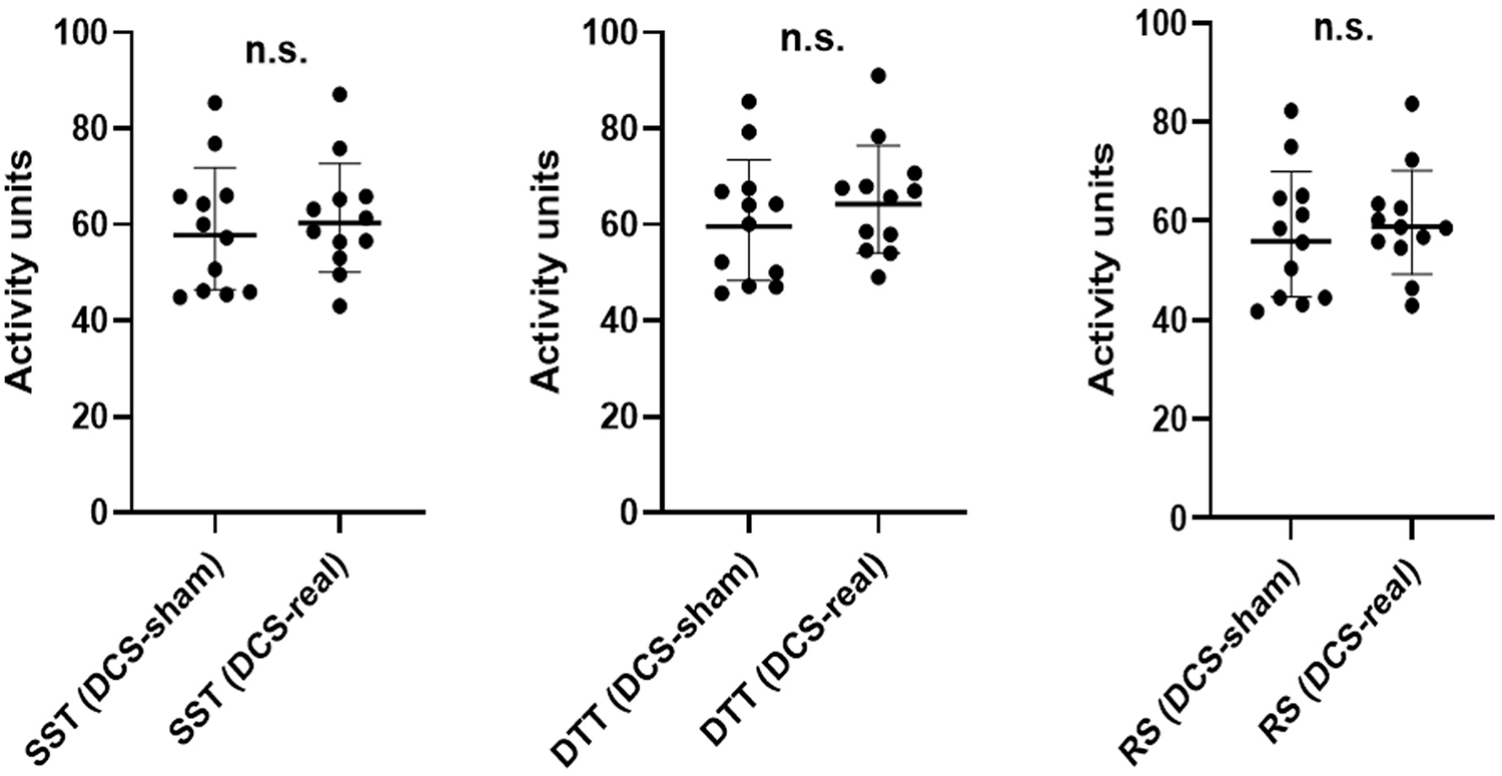
Dot plots representing hemoencephalography responses for the cognitive behavioral assessments and resting state (SST: Spatial span task; DDT: Double trouble task; RS: Resting state).

Upon resting state, similar results were obtained and revealed no statically significant difference between participants' performance in the sham- and real-DCS sessions (*p* > 0.05) (Fig. 4).

### Hand Grip Strength Test

**Figure 5:**
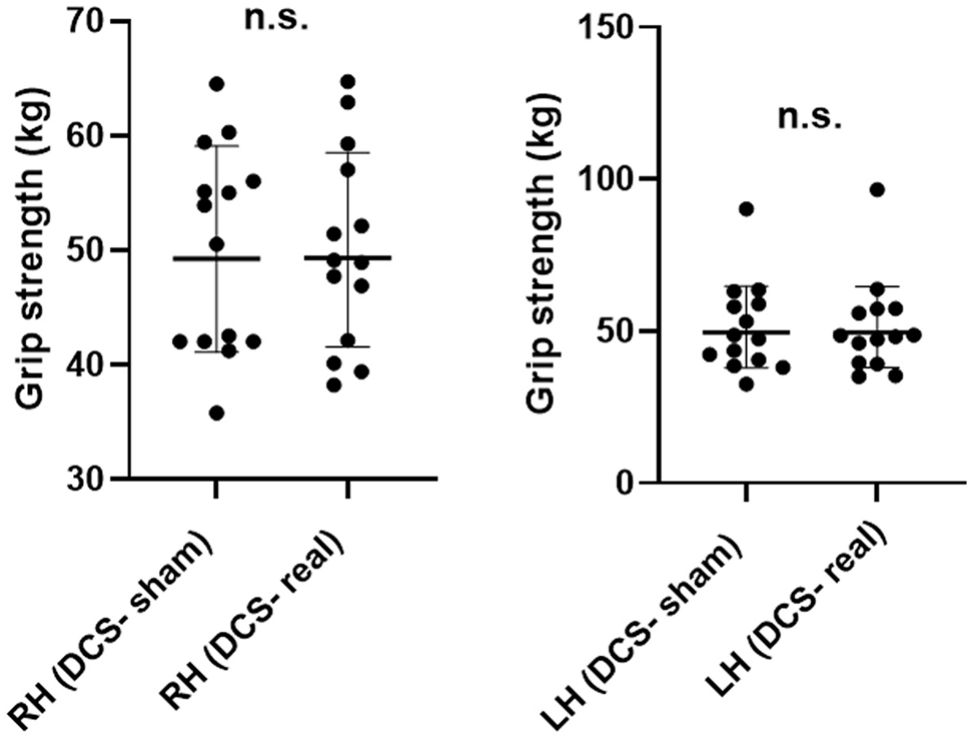
Dot plots representation of differences in grip strength of participants between real and sham DCS (RH: Right hand; LH: Left hand).

The analysis of participants’ results in the Hand Grip Strength Test recorded by our digital dynamometer revealed no statically significant difference between the sham- and real-DCS sessions. These analyses yielded the same results both for the right and left hands (*p* > 0.05) (Fig. 5).

## Discussion

The functional impact of tDCS and tsDCS might be attributed to an altered spontaneous neural activity and membrane potentials of the cortical and corticomotoneuronal cells, respectively^18^. Based on the earlier research findings on tDCS or tsDCS and their effects on motor performance, either of such modalities are found to be effectively enhancing the motor outcome in healthy individuals and professional athletes^19–21^.

However, these modalities have not yet been examined in boxers neither alone nor in combination. Building upon the existing evidence, the present research hypothesized the efficacy of this combination approach in boxers and evaluated the outcome measures. Nevertheless, the questions whether each modality on its own, is going to be effective, or the combination effect is additive vs. synergistic, are yet to be explored and was not the focus of the present research.

A multi-arm randomized sham-controlled designed study (sham tDCS-sham tsDCS/sham tDCS-real tsDCS/real tDCS-sham tsDCS/real DCS-real tsDCS) will certainly be needed to address the above open questions and thus not having addressed the above can be considered as a potential strength-in-the-making to our study. One of the key limitations to our work was a relatively small sample-size that hindered us from conducting a multi-arm randomized controlled trial at this stage.

Taken together, the present investigation comparatively analyzed the motor outcome in boxers in a single arm study design through random sequential stimulation sessions by real/sham combination of tDCS and tsDCS (Fig. 1A), and could confirm the efficacy of this intervention based on the initial hypothesis.

Our study findings suggest that the simultaneous stimulation of motor cortex and spinal cord significantly improves selective attention and reaction speed in experienced boxers. Neuromodulation and brain stimulation techniques have been used in recent years to improve athletic performance; however, few systematic studies^2,7,22,23^ have analyzed these new techniques. Most available sport studies have investigated the endurance of athletes^5,23^, and no researcher seems to have investigated the effects of brain stimulation on the accuracy, acceleration, and visuospatial memory of experienced boxers. Considering the critical role of the brain and spinal cord in athletic performance^24^, this study examined whether the simultaneous stimulation of the spinal cord and primary motor cortex (of the hand area) improves cognitive processing and performance of boxers. The effect of this stimulation on athletes’ cognitive function (visuospatial working memory and selective attention) was also investigated.

Furuya et al*.*^25^ concluded that the maximum capacity of professional pianists (as a typical example for motor learning) is limited and cannot be increased with tDCS technique. Despite their findings, we enrolled professional boxers to determine whether there is a similar capacity limit in professional boxers or CNS stimulation can help them achieve even higher levels in their athletic performance.

Studies have shown that extreme sports can cause central fatigue and reduced spinal reflexes^26–28^. Spinal cord stimulation over the T11 and T12 vertebrae has been shown to improve an individual’s explosive vertical jump performance^21^. The precise mechanism of tsDCS stimulation has not yet been discovered; however, tsDCS has been found to modulate spinal cord function, and thereby improve motor activity by facilitating the function of spinal cord motor neurons^11^. TsDCS also inhibits the transmission of pain signals to the spinal cord surface, and this may also be effective in competitive sports that are accompanied with severe muscle aches^11^. Although few studies have investigated the effect of tsDCS on healthy individuals, many studies have examined the effect of tDCS on the motor functions of healthy people. For example, tDCS has been shown to reduce perceived fatigue levels and increased endurance levels in athletes by modulating the function of M1^6,22^. Additional, the intervention has also been proposed to enhance motor learning in athletes^29^, and improve cognitive function in healthy individuals^30^.

Nevertheless, this stimulation may also impair an individual’s cognitive functions. Since cognitive functions are extremely vital for the athletes^14,15^, studies need to also rule out any adverse or untoward effects of neurostimulation on the athletes’ cognitive aptitude. Therefore, we used the CBS-CP to determine any possible positive or negative effects of our brain stimulation protocol on the participants’ cognitive functions. This platform has three sections of reasoning, memory, and verbal skills (cambridgebrainsciences.com). One task was selected from each section to examine the impact of tDCS on various cognitive aspects. We showed that the proposed tDCS montage has no negative effect on the participants’ cognitive functions. The maximum effect of a tDCS session lasts for approximately 1 h^31^; therefore the researchers had to limit the cognitive assessments to 3 tasks. Further assessments are needed to examine how this type of stimulation affects other cognitive functions.

With respect to the duration of the stimulation sessions, the issue of dose–response in DCS has been a subject for some earlier research works. Studies have indicated that the effect of at least 10 min of brain stimulation would last for an hour after the intervention^32^. Nitsche et al*.* showed that the effects of a tDCS session (2 mA, 13 min) continued to remain for 150 min^32^. In addition, a study on cyclists and one of our earlier research works on bodybuilders indicated the effectiveness of 13 min of stimulation in enhancing athletic performance^6,22^. As such, we considered ‘13 min’ as an optimal stimulation duration already examined. It is worth noting that the length of stimulation was 20 min in most studies. We considered a shorter length of stimulation as it could be more convenient before sport competitions.

An increase in corticospinal excitability and stimulation may have played an important role in increasing reaction speed. Research has shown that tDCS stimulation over M1 can stimulate corticospinal output^32,33^. It can probably be concluded that stimulation of the motor cortex stimulates this area and increases corticospinal output in sports tasks^34^. M1 stimulation probably led to an increase in reaction speed by using more motor units. The use of NMDA receptor antagonists (such as dextromethorphan) has been shown to reduce the effect of tDCS^35^. According to studies, in addition to the reaction speed, tDCS may also increase the punching force. Anodal tDCS over the cortex can also improve muscular power by using more motor units probably due to the acute corticospinal responses^6,36,37^. In addition, studies have shown that tsDCS can improve motor reflexes^38^, which can also enhance the abilities of boxers. TsDCS probably accounts for a large part of improvement in reaction speed; however, since selective attention is related to higher cortical function, tDCS technique probably plays a more important role in improving this ability.

Due to the limited number of professional boxers, this study investigated the effects of both tDCS and tsDCS techniques on boxers; however, these techniques can be separately investigated in future studies. Researchers can also examine the effects of these methods on the punching force of boxers.

With respect to the keywork “neuro-doping”, is appears that a whole new perspective of research and ethical standards need to be formulated. Today, research in sport neuroscience is on the verge of delineating the risks, concerns, and benefits of such an approach in real life.

The word ‘doping’ broadly corresponds to the use of illegitimate means, specifically drugs, to enhance performance in athletes. Meanwhile, the arguable use of merging neurotechniques to stimulate the brain and nervous system in healthy people has brought about some interests and raised some concerns. There are some reports on the impact of electric (tES) or repetitive transcranial magnetic stimulation (rTMS) to enhance physical and mental performance in professional athletes^2^.

The hypotheses that either tES or rTMS are efficient in enhancing athletic performance indicators, including the shortening of reaction times to visual, auditory and touch stimuli, reducing tremor, and enhancement in the acquisition of complex motor skills, have been tested in some earlier investigations. Yet, head-to-head comparative studies on the effects of rTMS *vs.* tES seem to be lacking. An existing body of research postulates that brain stimulation through rTMS and tES speeds up motor learning and improves motor skills in sport activities. However, the precise mechanisms involved in the above need to be scrutinized in extended lines of research^39^.

In addition, the ethical analysis of the use, or possible use, of neuro-doping in sport is a neglected subject. In that realm, the question whether tES or rTMS in healthy professional athletes should be added to World Anti-Doping Agency’s (WADA) prohibited list or not, is still open to debate. Furthermore, whether ‘the use of neuro-doping is unfair’ depends not only on the overall long-term safety as well as the accessibility and ease of use among athletes, but also the pending rules and regulations to be laid down by WADA. Collective analyses in a recent publication have proposed that at present ‘neuro-doping’ cannot be considered a threat to the integrity of sport. Albeit, the above reassurance largely depends on the fact whether in the future, neuro-stimulation techniques become among effective performance-enhancing means in sport^40,41^.

## Conclusion

The present report generally suggests that simultaneous anodal tDCS over M1 and spinal cord may help professional boxers improve their overall performance. This study prepares the ground for designing CNS stimulation protocols to enhance essential athletic factors including accuracy and reaction speed. Considering the effect of tsDCS on spinal reflexes, hypothetically is may improve motor cortex stimulation and enhance athletic performance in a synergistic way. Given the positive effect of this montage on experienced boxers’ athletic performance, it can probably influence an athlete’s success in intense professional competitions.

## Data Availability

The authors have shared “minimal data set” for the present submission as per the journal’s policy for data availability.
